# Comparisons of the Factor Structure and Measurement Invariance of the Spence Children’s Anxiety Scale—Parent Version in Children with Autism Spectrum Disorder and Typically Developing Anxious Children

**DOI:** 10.1007/s10803-017-3118-0

**Published:** 2017-04-09

**Authors:** Magdalena Glod, Cathy Creswell, Polly Waite, Ruth Jamieson, Helen McConachie, Mikle Don South, Jacqui Rodgers

**Affiliations:** 1 Institute of Neuroscience, Newcastle University, Level 3, Sir James Spence Institute, Royal Victoria Infirmary, Queen Victoria Road, Newcastle upon Tyne, NE1 4LP UK; 2 Anxiety and Depression in Young People (AnDY) Research Clinic, School of Psychology and Clinical Language Sciences, University of Reading, Earley Gate, Reading, RG6 6AL UK; 3 School of Psychology, Newcastle University, 4th Floor, Ridley Building 1, Queen Victoria Road, Newcastle upon Tyne, NE1 7RU UK; 4 Institute of Health and Society, Newcastle University, Level 3, Sir James Spence Institute, Royal Victoria Infirmary, Queen Victoria Road, Newcastle upon Tyne, NE1 4LP UK; 5 Department of Psychology, Brigham Young University, 245 TLRB, Provo, UT 84602 USA; 6 Present Address: Greater Glasgow and Clyde NHS, Glasgow, Scotland, UK

**Keywords:** Anxiety, SCAS-P, Measurement invariance, Autism spectrum disorder, Anxiety disorders

## Abstract

The Spence Children’s Anxiety Scale—Parent version (SCAS-P) is often used to assess anxiety in children with autism spectrum disorder (ASD), however, little is known about the validity of the tool in this population. The aim of this study was to determine whether the SCAS-P has the same factorial validity in a sample of young people with ASD (n = 285), compared to a sample of typically developing young people with anxiety disorders (n = 224). Poor model fit with all of the six hypothesised models precluded invariance testing. Exploratory factor analysis indicated that different anxiety phenomenology characterises the two samples. The findings suggest that cross-group comparisons between ASD and anxious samples based on the SCAS-P scores may not always be appropriate.

## Introduction

Anxiety is a common health concern in children with autism spectrum disorder (ASD), affecting between 11–84% (White et al. 2009) compared to 3–24% of typically developing children (Green and Ben-Sasson 2010). A meta-analysis (Van Steensel et al. 2011) reported that nearly 40% of individuals with ASD display clinical levels of anxiety and anxiety is one of the most common comorbid psychiatric disorders in children with ASD (Simonoff et al. 2008). Furthermore, anxiety problems can lead to increased maladaptive behaviour (Kim et al. 2000), unemployment, and chronic mental health difficulties among young people with ASD (Farrugia and Hudson 2006). Although the recognition of anxiety problems in ASD has a long history, starting as early as with the first description of autism by Kanner (1943), the assessment and treatment of anxiety in individuals with ASD has only recently begun to receive the empirical attention it needs and deserves (Rodgers et al. 2012; White et al. 2009). There remains a critical need for the development of valid and reliable assessment measures to accurately identify anxiety in children and young people with ASD.

MacNeil, Lopes and Minnes (2009) reported that young people with ASD have higher levels of anxiety than typically developing children and comparable levels of anxiety to typically developing clinically anxious children. As is the case among typically developing populations, some forms of anxiety appear to be more common than others in children with ASD (Van Steensel et al. 2011); for example specific phobias are more common than separation anxiety and panic disorder. Sukhodolsky et al. (2008) report the prevalence rates for specific phobias, separation anxiety and panic disorder in children with ASD aged between 5 and 17, as 31, 10.5 and 0.0%, respectively. Rates reported for obsessive–compulsive disorder (OCD), social anxiety disorder (SAD) and generalised anxiety disorder (GAD) vary widely across studies in ASD (2.6–36.7% for OCD; 0.5–27.3% for SAD; and 1.2–45.2% for GAD; Van Steensel et al. 2011). Understanding this variability is important and it may be that it is influenced by a number of factors, including the specific challenges of accurately measuring anxiety in ASD.

The presentation of anxiety in children with and without ASD shares some common features, such as social fears that are characteristic of social phobia (Settipani et al. 2012). However, there may also be some unique aspects of anxiety in ASD, for example there is evidence for an association between anxiety and both sensory over-responsivity (Ben-Sasson et al. 2008; Green and Ben-Sasson 2010) and impairment in social functioning in ASD (Bellini 2004, 2006). Thus, young people with ASD may be predisposed to anxiety as a result of a range of ASD-specific factors. Furthermore, there is also evidence that anxiety can exacerbate some of the features of ASD, such as repetitive behaviours (Sofronoff et al. 2005). Kanner (1943) observed that “an insistence on sameness, and the repertoire of fixed behaviours and routines” appeared to have a strong association with anxiety (Kanner 1943, as cited in; Gillot et al. 2001, p. 277). Features of ASD and symptoms of anxiety may however overlap and prove difficult to delineate (Gjevik et al. 2010). For example, repetitive behaviours seen in ASD can be difficult to differentiate from the compulsive behaviours found in OCD (Zandt et al. 2009). Also atypical anxiety symptoms have been reported to be associated with ASD symptomatology, strengthening the overlap and relationship of anxiety and repetitive and restricted behaviours in ASD (Kerns et al. 2014). Furthermore, Mikita et al. (2016) suggested putative links between predisposing ASD traits and subsequent anxiety responses, possibly underpinned by a distinct pathophysiological mechanism. The authors indicated a possibility of distinguishing a distinct nosological category of individuals with ASD and comorbid anxiety that should be researched in its own right. That highlights the need for measures that include anxiety-related items that are specific to the phenomenology of anxiety in ASD (Rodgers et al. 2016). Rodgers and colleagues (2016) have recently developed the first autism-specific anxiety scale (ASC-ASD) with evidence of good reliability and validity.

Generally, the assessment of anxiety in ASD has relied on measures originally validated for use in typically developing populations (White et al. 2009). Given the distinct challenges of measuring anxiety in ASD, the precision of these instruments has been called into question. Van Steensel, Deutschman and Bögels (2013) evaluated the parent-report Screen for Child Anxiety-Related Emotional Disorders (SCARED-71; Bodden et al. 2009) for use in ASD. They reported that although psychometric properties of the measure were comparable for ASD and anxiety-disordered groups, alternative cut-off scores were recommended for young people with ASD. White, Schry and Maddox (2012) provided mixed evidence for the reliability and validity of both the Multidimensional Anxiety Scale for Children (MASC) and the Child and Adolescent Symptom Inventory-4 ASD Anxiety Scale when used with adolescents diagnosed with high functioning autism. The authors found that the measures had acceptable internal consistency, and there was evidence of discriminant validity, however,the youth self-report was found to have a questionable validity. Kaat and Lecavalier (2015) evaluated the self- and parent-reported revised child anxiety and depression scale (RCADS) and a more recent version of the MASC among youth with ASD and raised some concerns regarding the construct validity of anxiety in ASD as measured by these scales. More concerns were particularly raised about the interpretation and validity of child/youth self-report anxiety screening measures in the ASD group (Mazefsky et al. 2011; White et al. 2012). Moreover, acceptable internal consistency, modest convergent validity, and questionable divergent validity in separating anxiety from attention problems in ASD on the RCADS suggested that more convincing evidence is needed to use the tool in ASD (Sterling et al. 2015).

The Spence Children’s Anxiety Scale-Parent (SCAS-P; Spence 1998) is frequently used in ASD research (Chalfant et al. 2006; McConachie et al. 2014; Rodgers et al. 2012; Russell and Sofronoff 2005; Sung et al. 2011). The SCAS-P is a parent-completed questionnaire for assessing the severity of a range of anxiety symptoms. It has been reported to be a reliable and valid tool for screening anxiety symptoms in typically developing children (Nauta et al. 2004). The parent-report measure also has high correspondence with the well-validated self-report Spence Children’s Anxiety Scale (SCAS; Nauta et al. 2004). Russell and Sofronoff (2005) found both parent and child versions of the questionnaire had high internal reliability in ASD samples. Findings from the recent psychometric work done on the questionnaire showed that there was overall moderately good agreement between caregivers’ and ASD children’s reporting of anxiety symptoms using the SCAS-P and the SCAS (Magiati et al. 2014); and suggested that the SCAS-P could be a useful screening tool for anxiety disorders in ASD (Zainal et al. 2014). A recent systematic review of outcome measures used in anxiety intervention studies for high-functioning children with ASD suggested that the SCAS-P, its revised version, the RCADS, and the SCARED had the most robust measurement properties (Wigham and McConachie 2014). However, there was little or no evidence for some aspects (e.g. responsiveness to change and content validity). Little is yet known about the reliability or validity of the SCAS-P as a measure of anxiety in children with ASD.

It remains unclear whether the SCAS-P measures the same constructs in ASD as it does in typically developing clinically anxious children (without ASD). Moreover, the subsequent question of whether this instrument measures the construct in the same way, should also be addressed to enable valid comparisons of observed scores across groups to be made. Further investigation is required to enable confidence that the scale functions in the same way across clinical groups.

In order to establish whether a given measure of a particular latent construct (such as anxiety) performs similarly across the groups, it has been suggested that measurement invariance should be first performed (Vandenberg and Lance 2000). Only then can meaningful comparisons between groups be made as measurement invariance analysis indicates whether the instrument measures the same construct in the same way across different populations or groups (Millsap and Kwok 2004).For example, Garnaat and Norton (2010) assessed measurement invariance of the Yale-Brown obsessive compulsive scale across four racial/ethnic groups (namely, White, Black, Asian, and Hispanic). They found generally stable properties although highlighted some concern that some scales may underestimate diagnosis of OCD in Black groups.

To our knowledge, there has been no attempt to use measurement invariance to compare separate clinical groups. The aims of this study were two-fold. Firstly, to determine the factor structure for the SCAS-P in a sample of young people with ASD and to compare it with the factor structure derived from a sample of clinically-anxious young people without ASD, and in the combined sample to ensure adequate fit to consider invariance. Secondly, to use measurement invariance techniques to determine whether SCAS-P items function in the same way in children with ASD and anxious children without ASD, in order to establish whether cross-groups comparisons using the SCAS-P are appropriate and meaningful. Due to concerns raised about both validity and interpretation child/youth self-report anxiety measures in the ASD group, the parent version of the SCAS was the main focus of this study.

## Methods

### Measure

The Spence Children’s Anxiety Scale-Parent Version (SCAS-P; Spence 1998) is a 38-item checklist, where parents rate the frequency of occurrence of anxiety symptoms on a four-point Likert-type scale, ranging from 0 (never) to three (always). Thus, higher scores indicate increased levels of anxiety. SCAS-P mean norms for the total score in healthy children and young people range between 11.8 and 16, increasing to 30.1 to 33 in anxiety disordered children and adolescents (Nauta et al. 2004). The scale provides a total anxiety score as well as six subscale scores developed to reflect symptoms characterized by the Diagnostic and Statistical Manual of Mental Disorders, Fourth Edition (DSM-IV): panic and agoraphobia; separation anxiety; social phobia; physical injury fears; OCD, and GAD. The proposed 6-factor structure has been supported by confirmatory factor analyses (Nauta et al. 2004). The SCAS-P is reported to have satisfactory to excellent reliability and shows acceptable validity for anxious children (Nauta et al. 2004).

### Participants

The study involved analysis of archival data pooled from several different settings.

#### ASD Sample

This group consisted of parents of 285 children and adolescents with ASD, recruited from four sources. Most children and adolescents (211participants, 181 male, mean age in months = 147.95, SD = 24.1; range 8–16 years old) were seen by health and education teams in the North East of England, recruited through Daslne (Database of Children with Autism Spectrum Disorder Living in the North East); McConachie et al. 2009). The second group consisted of those who took part in the Beating Anxiety Together (BAT) project (McConachie et al. 2014), an intervention programme created for children and adolescents with ASD who also had comorbid high anxiety (21 participants, 20 male, mean age in months = 137.05, SD = 16.22; range 8.92–13.58 years old). The third group (19 participants, 16 male, mean age in months = 139.74, SD = 29.66; range 8.83–15.58 years old) took part in the UK part of the ‘Fun and Games’ study investigating decision making styles used by individuals with ASD (Boulter et al. 2014; South et al. 2014). Finally, 34 participants (29 male, mean age in months = 139.50, SD = 35.90; range 7.05–17.09 years old) were recruited for a study based at Newcastle University, UK, investigating the relation between executive functioning, sensory processing and anxiety (Darus, unpublished PhD). All children were diagnosed through a multidisciplinary team assessment following the guidelines of the UK National Autism Plan for Children (Le Couteur 2003). All met criteria for ASD on the Autism Diagnostic Observation Schedule (ADOS; Lord et al. 2000), administered and rated from video by trained raters who maintained over 80% agreement with consensus ADOS ratings. In all cases, one parent completed the SCAS-P, reporting on their child’s symptoms of anxiety. The mean of the SCAS-P total score was 33.85 (SD = 19.65) in the ASD sample. The means of subscales were as follow: Panic attack and agoraphobia: 4.75 (SD = 4.48), separation anxiety: 5.97 (SD = 4.12), physical injury fears: 4.87 (SD = 3.24), social phobia: 7.26 (SD = 4.90), obsessive compulsive: 5.00 (SD = 3.93), generalized anxiety disorder: 6.00 (SD = 3.76).

#### Anxious Sample

The anxiety-disorder group included data from parents of non-ASD, clinically anxious children and adolescents referred to the Berkshire Child Anxiety Clinic, University of Reading, UK. SCAS-P data from this sample was collected from parents of 224 (150 male) children and adolescents with a mean age in months of 144.92 (SD = 32.82, range 8–17 years old). The mean total score of the SCAS-P was 38.47 (SD = 17.02). The means of subscales were as follow: panic attack and agoraphobia: 5.44 (SD = 4.93), separation anxiety: 7.56 (SD = 4.26), physical injury fears: 4.60 (SD = 2.77), social phobia: 9.11 (SD = 4.35), obsessive compulsive: 3.98 (SD = 3.57), generalized anxiety disorder: 7.78 (SD = 3.63).

For that sample, on receipt of referral, parents completed a number of screening questionnaires to ensure that anxiety was the primary concern. This screening included the Social Communication Questionnaire to screen for characteristics of ASD (Rutter et al. 2003). Where children scored above clinical cut-offs (≥15) further investigations were conducted to ensure that children did not meet criteria for ASD. All children met diagnostic criteria for a primary anxiety disorder as established by the Anxiety Disorders Interview Schedule for DSM-IV structured interview (ADIS-C/P; Silverman and Albano 1996), a structured diagnostic interview with well-established psychometric properties (Silverman et al. 2001). Where children met symptom criteria for a diagnosis they were assigned a clinical severity rating (CSR) ranging from 0 (complete absence of psychopathology) to eight (severe psychopathology). As is conventional, overall diagnoses and CSRs were assigned if the child met diagnostic criteria on the basis of either child or parent report, and the higher CSR of the two was taken. Only those who met symptom criteria with a CSR of four or more (moderate psychopathology) were considered to meet diagnostic criteria. Assessors (psychology graduates) were trained on the administration and scoring of the ADIS and ADIS-C/P through verbal instruction, listening to assessment audio-recordings and participating in diagnostic consensus discussions. The first 20 interviews conducted were then discussed with a consensus team, led by an experienced diagnostician (Consultant Clinical Psychologist). The assessor and the consensus team independently allocated diagnoses and CSRs. Following the administration of 20 child or 20 parent interviews, inter-rater reliability for each assessor was checked, and if assessors achieved reliability of at least 0.85 they were then required to discuss one in six interviews with the consensus team (to prevent inter-rater drift). Overall reliability for the team was excellent. As different assessors interviewed the parent and child simultaneously reliability figures for parent and child report were calculated separately. Reliability for presence or absence of diagnosis on the ADIS-C/P was kappa = 0.98 (child report), 0.98 (mother report); and for the CSR intra-class correlation = .99 (child report), 0.99 (mother report). Reliability for presence or absence of maternal diagnosis on the ADIS was kappa = 0.97; and for the CSR intra-class correlation = .99. Primary anxiety diagnoses for the sample were generalised anxiety disorder (n = 55), social phobia (n = 61), separation anxiety disorder (n = 40), specific phobia (n = 41), OCD (n = 3), agoraphobia without panic disorder (n = 9), anxiety disorder not otherwise specified (ADNOS; n = 5), and panic disorder (n = 10).

### Analysis Plan

All analyses were conducted using SPSS 21 (IBM SPSS Statistics for Windows, Released 2012) and AMOS 21.0.0 (Arbuckle 2012) software programs. There were missing values only in our anxious sample. There were no particular patterns in the missing data, allowing the data to be treated as missing completely at random. Participants with over 20% of missing item level data were removed (n = 3) to minimalize randomness in our dataset. For the remaining participants the maximum likelihood estimation method of data imputation was used to complete the dataset.

#### Confirmatory Factor Analysis

In order to determine the factor structure of the SCAS-P, a confirmatory factor analysis (CFA), using structural equation modelling, in AMOS, was conducted with data from the anxious and ASD samples separately, and then in the combined sample, in order to determine the best-fitting factor structure and assess invariance. Six hypothesised models were tested subsequently. Five were the DSM-IV-based symptom models suggested by Nauta et al. (2004) including: (1) one factor, (2) six uncorrelated factors, (3) six correlated factors, (4) six correlated factors and one higher order factor, and (5) five correlated factors and generalized anxiety as one higher-order factor. For anxiety disordered children, as suggested by Nauta et al. (2004), support was found for six intercorrelated factors (separation anxiety, generalized anxiety, social phobia, panic/agoraphobia, OCD, and fear of physical injuries) and a model with generalized anxiety as the higher order factor for the other five factors. There is no support in the literature that either of the models would fit ASD sample. The sixth model tested in this study was based on work done by Jamieson et al. (unpublished thesis, 2012) who suggested that five correlated factors (with GAD subscale excluded) might be the best-fitting factor structure for children and adolescents with ASD. All models were tested in order to establish whether any of the hypothesised models would provide the fitting factor structure for either of the samples.

Model fit was evaluated using established recommendations identified as “best behaved” on the basis of previous research (Brown 2006, p. 85; Hu and Bentler 1999). For example, we followed recommendations that χ^2^/*df* ratio (Bryant and Yarnold 1995) should be close to zero and that root mean square error of approximation (RMSEA) values close to 0.06 represent good fit (Hu and Bentler 1999), whilst values less than 0.08 are indicative of acceptable fit, and values between 0.08 and 0.10 represent poor model fit (Browne and Cudeck 1993). It is recommended that the comparative fit index (CFI) is greater than 0.95, but a level greater than 0.9 being acceptable (Hu and Bentler 1999). It is also recommended that the Tucker-Lewis index (TLI) is greater than 0.90 to demonstrate good fit (Brown 2006). The non-significant Chi square (χ^2^) statistic (Brown 2006) may be used an indicator of fit, however, because it is greatly influenced by sample size (Stevens 2002), we did not use in isolation from other recommended goodness of fit indices. The Chi square difference test was also used to compare competing models.

#### Measurement Invariance

The measurement invariance technique can be implemented by running a multi-group analysis of the factor structure that underlies the data of two groups (Byrne and Campbell 1999). The following sequence of four nested models is usually tested (see Cheung and Rensvold 2002; Schmitt and Kuljanin 2008): configural invariance; metric invariance; scalar invariance; and residual (uniqueness) invariance. In the configural invariance model, the same factor structure is implied for two or more groups of participants entered into the analysis. The values of the parameters (i.e. factor loadings, intercepts, residual variances) may vary across the groups, as no equality constraints are imposed. In the metric invariance model whether the values of the factor loadings are the same across groups is tested; hence item loadings are constrained to be equal across groups. Scalar invariance tests latent factor mean differences across groups and is evaluated by constraining the intercepts of measures to be the same across groups. In the residual model items unique variances are constrained to be equal across the two (or more) comparison groups. As suggested by Chen (2007), suggested differences in both CFI (delta CFI <0.01) and RMSEA (RMSEA <0.015) values were considered when comparing two nested models e.g. metric and scalar invariance.

## Results

### Preliminary and Descriptive Statistics

Examining the SCAS-P samples, anxious and ASD participants did not significantly differ on age. A significant difference was found for gender, with more female participants in the anxious sample. However, this difference represents the general sex ratio typical for the ASD population, with more males than females diagnosed with the condition (Werling and Geschwind 2013).

### Confirmatory Factor Analysis

In the anxious, ASD and combined (both anxious and ASD) samples, six models, including: (1) one factor, (2) six uncorrelated factors, (3) six correlated factors, (4) six correlated factors and one higher order factor, (5) five correlated factors and generalized anxiety as one higher-order factor, and (6) five correlated factors (with GAD subscale excluded), were tested. The goodness of fit indices are summarised in Table 1.

**Table 1:** Fit indices for six hypothesised models for the anxious, ASD and combined sample

Hypothesised Model:	χ²	df	χ²/df	*p*	CFI	TLI	RMSEA
Model 1: one factor							
ANX	2250.07	665	3.38	<0.001	.53	.50	.103
ASD	2428.89	665	3.65	<0.001	.66	.64	.097
Combined	3984.07	665	5.99	<0.001	.60	.58	.099
Model 2: six uncorrelated factors							
ANX	2171.05	665	3.27	<0.001	.55	.53	.101
ASD	2833.2	665	4.26	<0.001	.58	.55	.107
Combined	4173.27	665	6.28	<0.001	.58	.56	.102
Model 3: six correlated factors							
ANX	1685.40	650	2.59	<0.001	.69	.67	.085
ASD	1908.08	650	2.94	<0.001	.76	.74	.083
Combined	2855.66	650	4.39	<0.001	.73	.71	.082
Model 4: six correlated factors and one higher order factor							
ANX	1703.40	659	2.59	<0.001	.69	.67	.084
ASD	1937.68	659	2.94	<0.001	.75	.74	.083
Combined	2878.88	659	4.37	<0.001	.73	.72	.081
Model 5: five correlated factors and generalized anxiety as one higher-order factor							
ANX	1711.32	661	2.59	<0.001	.69	.67	.084
ASD	1941.39	661	2.94	<0.001	.75	.74	.083
Combined	2880.18	661	4.36	<0.001	.73	.72	.081
Model 6: five correlated factors (with GAD subscale excluded)							
ANX	1134.13	454	2.49	<0.001	.73	.70	.082
ASD	1257.59	454	2.76	<0.001	.79	.77	.079
Combined	1839.60	454	4.05	<0.001	.77	.75	.077

Recommended goodness of fit indices values demonstrating good model fit: χ2/*df* ratio close to zero, RMSEA <0.6, CFI >0.95 and TLI >0.9 (Brown 2006; Hu and Bentler 1999)

Overall, fit indices fell below the generally recommended ranges for good fit in each model. Due to poor models’ fit subsequent invariance testing was not conducted as there was not enough evidence to assess invariance.

### Post-hoc Analysis

Due to the poor model fit with any of the six hypothesised models, we investigated the factor structure of the SCAS-P in the anxious and ASD samples with exploratory factor analysis (EFA). Parallel analysis and Velicer’s minimum average partial (MAP) test were performed to determine the number of components in the factor analyses. These validated procedures are superior to the eigenvalues greater-than-one rule (O’Connor 2000). In the ASD and anxious sample parallel analysis indicated an eight factor solution. The MAP test indicated six factors in the ASD sample and seven factors in the anxious sample. When differences in test results emerge, optimal decisions should be made after considering the results of both analytic procedures bearing in mind that the MAP test tends to underextract the number of factors, whereas parallel analysis tends to overextract the number of factors (O’Connor 2000). In both the eight and seven factor solutions in the ASD sample and the eight factor solution in the anxious sample, one factor consisted only of two items. The six factor solution in the ASD sample and seven factor solution in the anxious sample were considered as the most optimal. Maximum Likelihood extraction with oblique rotation was used because high correlations between the components were found (above 0.4 and below −0.4 in both groups) (Tables 2, 3).

**Table 2:** Rotated factor loadings in exploratory factor analysis of SCAS-P in ASD sample

Item	Content	Communalities	Factor 1 R^2^ = 33.46 E = 12.72	Factor 2 R^2^ = 6.27 E = 2.38	Factor 3 R^2^ = 5.05 E = 1.92	Factor 4 R^2^ = 4.41 E = 1.67	Factor 5 R^2^ = 4.19 E = 1.59	Factor 6 R^2^ = 3.29 E = 1.25
4	Feeling afraid	.67	.**51**	−.12	−.04	.41	.05	.04
36	Bothered by bad or silly thoughts or pictures	.70	.**47**	−.32	−.11	.00	−.03	.30
17	Bad or silly thoughts	.54	.**44**	−.34	.05	.03	.04	.21
20	Something bad will happen to him/her	.59	.**41**	−.33	−.13	.15	.04	.05
26	What other people think of him/her	.74	.03	**−.84**	−.08	−.18	−.02	.08
9	Make a fool	.68	.02	**−.77**	−.06	.01	−.00	.07
10	Do badly at school	.61	.03	**−.71**	−.12	−.00	.03	−.02
6	Take a test	.47	.02	**−.59**	−.05	.20	.02	−.08
31	Talk in front of the class	.43	−.04	**−.54**	−.03	−.02	.21	.05
18	Heart beating really fast	.79	−.07	−.04	**−.91**	.08	−.04	−.09
32	Heart suddenly starting to beat too quickly	.78	−.03	−.01	**−.89**	.04	−.12	.09
12	Can’t breathe	.41	−.07	−.08	**−.53**	−.05	.13	.10
30	Becoming dizzy or faint	.42	−.00	−.08	**−.44**	.05	.20	.06
5	Own at home	.55	−.02	−.09	−.11	.**69**	−.01	−.06
8	Being away from parent	.57	−.10	−.14	−.10	.**59**	.05	.11
2	Dark	.43	.20	−.02	−.04	.**58**	.01	−.02
14	Sleep on his/her own	.39	.07	.06	−.09	.**58**	.08	−.05
38	Stay away from home overnight	.45	.01	−.09	.02	.**47**	.25	.06
19	Tremble or shake	.55	.28	.18	−.27	−.05	.**55**	−.02
25	Travel in the car, or on a bus or train	.46	−.07	−.09	−.16	.05	.**52**	.05
22	Feels shaky	.57	.26	−.03	−.18	−.11	.**51**	.13
21	Doctor or dentist	.29	−.04	−.11	.11	.12	.**45**	.06
28	Scared for no reason	.64	.30	.00	−.04	.12	.**45**	.24
27	Crowded places	.46	−.14	−.20	−.05	.14	.**40**	.18
35	Do some things over and over again	.60	−.10	−.05	.05	−.06	.04	.**80**
37	Certain things in just the right way	.55	.16	.02	−.04	.03	.03	.**65**
13	Keep checking	.49	.00	−.04	−.11	.01	−.02	.**64**
24	Think special thoughts to stop	.39	.16	.05	−.22	−.05	.07	.**43**
1	Worries about things	.56	.39	−.36	.04	.25	−.02	.12
3	Funny feeling in stomach	.37	.18	−.18	−.25	.22	.07	.00
7	Public toilets and bathrooms	.46	−.21	−.23	−.09	.24	.39	.01
11	Something awful will happen to someone in the family	.48	.21	−.33	−.11	.28	−.22	.22
15	School in the mornings	.34	.09	−.34	−.07	.05	.27	−.02
16	Dogs	.08	−.12	.10	.03	.21	.01	.14
23	Heights	.20	.15	.10	−.09	.27	.10	.09
29	Insects or spiders	.16	.02	−.12	.01	.22	.11	.11
33	Suddenly get a scared feeling	.56	.35	.02	−.22	.07	.26	.22
34	Small closed places	.25	−.07	−.06	−.05	.16	.33	.08

Loading derived from maximum likelihood estimation with oblimin rotation. Content—summarized items content. *E* Eigenvalue. Communalities reported are post-extraction. Reported R^2^ and E derived from unrotated factor solution. Bold loadings >|.40|

**Table 3:** Rotated factor loadings in exploratory factor analysis of SCAS-P in anxious sample

Item	Content	Communalities	Factor 1 R^2^ = 25.66 E = 9.57	Factor 2 R^2^ = 8.32 E = 3.16	Factor 3 R^2^ = 6.35 E = 2.41	Factor 4 R^2^ = 5.50 E = 2.09	Factor 5 R^2^ = 4.16 E = 1.58	Factor 6 R^2^ = 3.79 E = 1.44	Factor 7 R^2^ = 3.69 E = 1.40
32	Heart suddenly starting to beat too quickly	.71	.**86**	.10	−.06	.04	.02	.05	−.12
18	Heart beating really fast	.67	.**81**	−.02	−.04	.04	−.05	.06	.02
12	Can’t breathe	.63	.**75**	.07	.01	.09	.02	.00	.06
19	Tremble or shake	.51	.**51**	−.09	−.17	−.17	−.04	−.10	.16
22	Feels shaky	.51	.**42**	−.07	−.17	−.30	−.06	−.19	.17
9	Make a fool	.71	−.06	.**83**	−.09	−.01	−.07	−.02	−.08
26	What other people think of him/her	.69	−.03	.**83**	−.02	−.01	−.14	.06	−.05
10	Do badly at school	.59	.01	.**76**	.03	.04	−.01	.04	.06
31	Talk in front of the class	.41	.05	.**58**	−.04	−.15	.12	−.05	.02
6	Take a test	.37	.21	.**52**	.02	−.01	.09	−.03	.07
37	Certain things in just the right way	.84	−.01	−.12	**−.93**	.00	−.08	.01	−.06
35	Do some things over and over again	.56	.03	.07	**−.71**	−.02	.01	−.08	.02
13	Keep checking	.47	.24	.21	**−.48**	.15	−.00	.07	−.05
5	Own at home	.49	.07	−.14	−.06	.**58**	−.02	−.25	.02
14	Sleep on his/her own	.43	−.04	−.06	−.10	.**55**	−.10	−.19	.03
2	Dark	.36	−.04	.08	−.01	.**54**	−.15	−.02	.08
17	Bad or silly thoughts	.65	.04	.06	−.07	.03	**−.74**	−.02	−.00
36	Bothered by bad or silly thoughts or pictures	.66	−.04	−.05	−.19	.06	**−.74**	.02	.04
20	Something bad will happen to him/her	.52	.23	.13	.08	.16	**−.45**	−.05	.14
38	Stay away from home overnight	.53	−.02	.01	−.16	.19	.11	**−.65**	.09
8	Being away from parent	.54	.03	.08	−.05	.24	−.07	**−.61**	−.02
15	School in the mornings	.38	.01	.23	−.03	−.14	−.07	**−.48**	−.06
4	Feeling afraid	.48	.10	−.02	.03	.23	−.26	**−.47**	−.04
3	Funny feeling in stomach	.42	.17	.02	.07	−.11	−.15	**−.46**	.13
33	Suddenly get a scared feeling	.50	.15	−.03	−.05	−.15	−.30	**−.45**	.06
34	Small closed places	.47	.05	−.02	−.05	.08	.09	−.01	.**67**
25	Travel in the car, or on a bus or train	.39	.01	.10	−.04	−.14	−.07	−.19	.**48**
27	Crowded places	.45	.01	.19	−.07	−.19	−.14	−.18	.**42**
23	Heights	.21	.06	−.12	.02	.03	−.08	.12	.**41**
1	Worries about things	.44	.09	.32	−.05	.18	−.22	−.24	.04
7	Public toilets and bathrooms	.35	−.01	.20	−.05	.19	.18	−.25	.38
11	Something awful will happen to someone in the family	.47	.27	.12	.07	.31	−.28	−.20	.00
16	Dogs	.12	0.04	−.07	−.04	.30	.04	.15	.03
21	Doctor or dentist	.24	0.10	.09	.04	.10	−.08	−.04	.36
24	Think special thoughts to stop	.46	0.21	−.08	−.20	.19	−.11	.03	.35
28	Scared for no reason	.43	0.16	−.05	−.05	−.06	−.26	−.37	.14
29	Insects or spiders	.26	−0.08	.19	−.18	−.02	−.11	.26	.31
30	Becoming dizzy or faint	.43	0.39	.04	−.04	−.25	.15	−.03	.21

Loading derived from maximum likelihood estimation with oblimin rotation. Content—summarized items content. *E* Eigenvalue. Communalities reported are post-extraction. Reported R^2^ and E derived from unrotated factor solution. Bold loadings >|.40|

For both groups the social phobia factor was derived and was very similar to the original social phobia factor (Nauta et al. 2004), with only item seven (‘My child is afraid when (s)he has to use public toilets or bathrooms’) not loading onto that factor. Also an OCD factor was derived that was similar to the original suggested by Nauta and colleagues (2004), however it consisted of only four items in the ASD group and three items in the anxious group. For the ASD group the other four factors comprised mostly of items belonging to the OCD, GAD, panic attack and agoraphobia, and separation anxiety subscales. Interestingly, panic attack and agoraphobia items loaded on two different factors. One factor included four items (item 19 ‘My child suddenly starts to tremble or shake when there is no reason for this’, item 25 ‘My child feels scared if (s)he has to travel in the car, or on a bus or train’, item 27 ‘My child is afraid of being in crowded places (like shopping centres, the movies, buses, busy playgrounds)’ and item 28 ‘All of a sudden my child feels really scared for no reason at all’) grouped together with one GAD item (item 22 ‘when my child has a problem, (s)he feels shaky) and one physical injury item (item 21 ‘My child is scared of going to the doctor or dentist’). A second factor related to the majority of the physiological symptoms of anxiety (item 32 ‘My child’s complains of his/her heart suddenly starting to beat to quickly for no reason’, item 12 ‘My child complains of suddenly feeling as if (s)he can’t breathe when there is no reason for this’, item 30 ‘My child complains of suddenly becoming dizzy or faint when there is no reason for this’ and GAD item 18 ‘when my child has a problem, (s)he complains of his/her heart beating really fast’). These three items relate to physiological symptoms of panic experience, including the ability to recognise those symptoms (e.g. increased heart beat) and communicate those changes in the body functions to others.

A split in the original panic and agoraphobia factor was also found in the anxious sample. Some of the items loaded on to a physiological symptoms of anxiety factor (with additional items from the original GAD factor) while the other factor was more agoraphobia specific (e.g. item 34 ‘My child is afraid of being in small closed places, like tunnels or small rooms’). Also OCD items separated into two distinct factors in the anxious typically developing group, with one relating to compulsions (e.g. item 37 ‘My child has to do certain things in just the right way to stop bad things from happening’), the other to obsessive thoughts (e.g. item 17 ‘My child can’t seem to get bad or silly thoughts out of his/her head’). Another factor that was indicated for the anxious group comprised of various separation anxiety, GAD and panic attack and agoraphobia items (e.g. item 33 ‘My child worries that (s)he will suddenly get a scared feeling when there is nothing to be afraid of’). The last factor consisted of two separation anxiety items (item five ‘My child would feel afraid of being on his/her own at home’ and item 14 ‘My child is scared if (s)he has to sleep on his/her own’) and one physical injury fears item (item two ‘My child is scared of the dark’). Items from across a range of the original subscales loaded on to the other factors in the anxious sample, with factor four including items ranging from separation anxiety to being scared of darkness, and factor five including items related to anxious thoughts and factor seven encompassing specific phobias.

## Discussion

The first aim of this study was to determine the factor structure for the SCAS-P in a sample of young people with ASD and to compare it with the factor structure derived from a sample of clinically-anxious young people without ASD, and in the combined sample to ensure adequate fit to consider invariance. However, due to poor model fit and inability to find an adequate baseline model for further between-group model testing, measurement invariance analyses could not be performed. Inability to find a model with a fixed number of factors in each group for the measure that has an established factor structure for use with typically developing samples was an unexpected outcome. Similarly, White et al. (2015) could not pursue the multigroup invariance factor analysis on the MASC parent version (but could on the MASC self-report), because the CFA undertaken on the typically developing anxious youth did not confirm the conventional MASC-P structure. It is important to bear in mind that parents might not always be aware of all anxiety-related behaviours that children exhibit, unless they verbalize their subjective and individual experiences. It is likely, particularly for our ASD sample, that parents were not aware of some of the symptoms or their severity and frequency. The reason why we could not find the baseline model of the SCAS-P in the anxious sample is unknown.

Using EFA, a six-factor model was established for the ASD sample, and a seven-factor model was found to describe the anxious sample best. The findings here for both groups differ from the SCAS-P factor structure suggested by Nauta et al. (2004), who found that six correlated factors fit the data obtained from the parents/caregivers of anxiety-disordered children best. Indeed, for the clinically anxious group we only found partial support for the panic attack and agoraphobia, OCD and social phobia factors. However, even within these factors some anomalies were found. Even less support for the original factor structure of the SCAS-P was found in the ASD sample.

The study showed limited support for the original factor structure of the SCAS-P. It is a novel, inconsistent with previous emotional functioning and personality literature (e.g., Hoelzle and Meyer 2009; Hopwood and Donnellan 2010; O’Connor 2002) finding. Some concerns, however, have been raised previously with regards to the validity of the SCAS-P, particularly of the GAD subscale for use with typically developing children. Spence et al. (2001) argued that this sub-scale could indicate more negative affect and autonomic responding than generalized anxiety, and found little support for a separate GAD-subscale. The content validity of the GAD subscale has been also questioned because it lacks overt reference to excessive worry (Chorpita et al. 1997), which is considered to be a central feature of GAD in childhood and adolescence. Our findings support these concerns, as a distinct GAD factor was not found in either our anxious or ASD samples. The physical injury fear factor was also not established for either of the samples. The reliability of the subscale, however, has been questioned previously, with unacceptable to questionable Cronbach’s alpha reported across community and clinical samples in various countries (Arendt et al. 2014; Whiteside and Brown 2008; Zainal et al. 2014). Although in the RCADS, a revised version of the SCAS-P, the measurement properties of GAD appeared to have improved (Wigham and McConachie 2014), evidence on psychometric properties of this tool remains patchy and requires further investigations.

According to our findings, further work is needed on the SCAS-P to establish its reliability and validity, particularly when used with the ASD population. Zainal and colleagues (2014) reported in their preliminary investigation, the SCAS-P might be a useful screening tool of anxiety in children with ASD when assessing elevated anxiety symptoms and relying on the total score. We suggest that a further caution is needed when using the tool to assess particular anxiety subtypes and make cross-groups comparisons between children with ASD and children diagnosed with anxiety disorder based on the SCAS-P scores. Although Wigham and McConachie (2014) reported that the SCAS-P was one of the tools to have the most robust measurement properties in comparison to other measures, there was lack of evidence for a number of reliability and validity characteristics of the questionnaire.

An important limitation to this study is that our anxious sample consisted of clinically referred individuals; and our ASD sample consisted of participants recruited to various studies, hence our sampling procedure might have impacted our findings. Further qualitative work is recommended to explore the validity of SCAS-P items in ASD samples. In line with other studies we recommend that the GAD and physical injury fears subscales require additional reliability and validity checks across clinical and community samples. Adaptation of the questionnaire is needed for reliable and valid use with ASD individuals. Qualitative interviews with parents should be conducted to better understand the context and particular situations in which caregivers base their answers.

## Conclusions

The SCAS-P has been developed and validated for use with typically developing youth. To use the scale as a reliable measure of anxiety in young people with ASD further work is needed. Researchers and clinicians should not rely solely on the scores obtained from the SCAS-P when assessing anxiety symptoms in individuals with ASD. Further and more systematic quantitative and qualitative research would be required to turn the SCAS-P into a robust measure of anxiety for use in ASD practice or research.
